# Lingual nerve injury after third molar removal: Unilateral atrophy of fungiform papillae

**DOI:** 10.4317/jced.51375

**Published:** 2014-04-01

**Authors:** Míriam Martos-Fernández, Alba de-Pablo-Garcia-Cuenca, Maria S. Bescós-Atín

**Affiliations:** 1 MD. Resident, Oral and Maxillofacial Surgery Department, Vall d’Hebrón Hospital. Barcelona, Spain; 2 MD. Assistant Surgeon, Oral and Maxillofacial Surgery Department, Vall d’Hebrón Hospital, Barcelona, Spain. Researcher of VHIR group; 3 MD, DDS, PhD. Head of Oral and Maxillofacial Surgery Department, Vall d’Hebrón Hospital. Barcelona, Spain. Oral and Maxillofacial surgeon at La Clinica Pilar, Barcelona, Spain. Researcher of the VHIR group

## Abstract

Background: Pain and sensory changes due to lingual nerve injury are one of the most common alterations that follow surgical removal of third molar. They are usually transient but other less common complications, such as the atrophy of fungiform papillae, have an uncertain prognosis. Case Description: We report a case of a 34-year-old woman who presented a unilateral lingual atrophy of fungiform papillae after third molar extraction accompanied by severe dysesthesia that altered her daily life significantly during the following months and how this complication evolved over time. We conducted a literature review on the different factors that can lead to a lingual nerve injury. Clinical Implications: The clinical evolution of temporary and permanent somatosensitve injuries is an important fact to take into consideration during the postoperative management because it will indicate the lesion prognosis.

** Key words:**Lingual nerve, third molar removal, somatosensitive alteration, papillae atrophy, permanent injury, temporary injury.

## Introduction

The surgical removal of the third molar, semi-erupted or included, is the most common dental procedure associated with lingual nerve injury (1). This lesion may involve temporary or permanent lingual sensory disturbances (anesthesia, paresthesia and/or dysesthesia) (2), sometimes accompanied by taste alterations in the anterior two thirds of the tongue causing problems like inability to chew properly or tongue biting (3). The incidence of temporary deficit is between 0-23% and permanent 0-8% ( Table 1), compared with temporary (0.4 to 8.4%) and permanent (<1%) lesion of the inferior alveolar nerve (4,5,6).

**Table 1:**
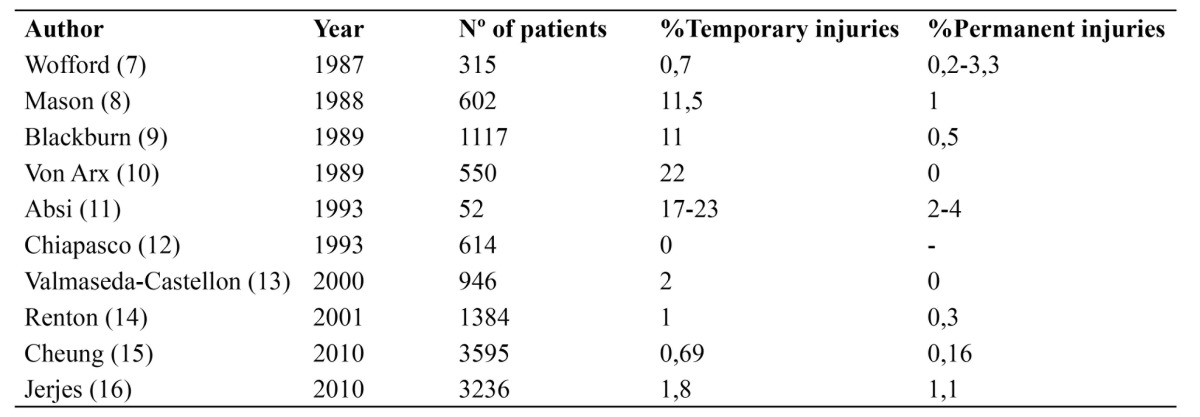
Literature review of the incidence of temporary and permanent lingual nerve deficit.

The lingual nerve, a branch of the mandibular nerve, provides somatosensory innervation of the lingual mucosa through its wide range of mechanosensitive, nociceptive and thermosensitives afferent fibers. Jointly with the chorda tympani nerve fibers, a branch of the facial nerve, it provides information to the anterior two thirds of the dorsum of the tongue and preganglionic parasympathetic innervation of submandibular and sublingual secretory glands (17). Its location is medial and anterior to the inferior alveolar nerve in its passage between the medial pterygoid muscle and the ramus of the mandible, where the fibers meet the chorda tympani nerve. It continues under the gingival sulcus of the lingual mucosa superficially to the surface of the gland and submandibular ganglion. It ends as a sublingual nerve located immediately below the tongue mucosa. Its proximity to the third molar lingual cortical plate, separated from the cortex only by the periosteum, and its variable anatomy (18) are the most important risk factors to be considered by the oral surgeon during surgical removal of the third molar.

This article presents a case of somatosensory lingual function loss accompanied by taste alterations and a unilateral atrophy of fungiform papillae as a complication of third molar extraction.

## Case Report

A 34 year old patient with no medical history was subjected to surgical removal of a third molar, located in the fourth quadrant in a disto-angular position (Winter’s classification). Before the procedure, an intraoral inferior alveolar nerve block with the usual technique (19) and a submucosal infiltration of buccal nerve were performed using two cartridges of 1.8 ml with epinephrine 40/0,005mg/ml using a 27G long needle without incidents. For a proper exposure of the tooth the standard Ward’s incision and a gutter in the disco-buccal bone were performed. Furthermore, it was necessary to perform a distal osteotomy and a lingual flap elevation without retraction. The total time of the intervention was 25 min. To close the wound, 3-0 silk suture was used.

**Figure 1:**
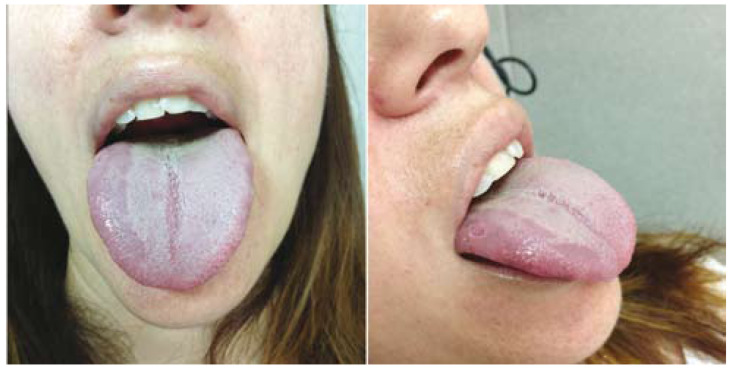
1st week after third molar removal. It shows a unilateral lingual atrophy of the fungiform papillae of right hemitongue accompanied by signs of recent bites.

During the immediate postoperative period the patient had pain and swelling limited to the right paramandibular area and it was reduced after oral NSAID analgesia (Ibuprofen). One week after removal the patient referred to altered taste of the anterior two thirds of the right hemitongue accompanied by intense dysesthesia and thermoreceptive and mechanoreceptive alterations. During physical examination a unilateral atrophy of fungiform papillae at the anterior two thirds of the right tongue side and signs of recent bites were observed (Fig. 1). The patient complained of severe pain in this area and subjective sensation of difficulty for proper diction but it was not clinically significant. No trigeminal or facial nerve alteration were observed. An analgesic treatment with Carbamazepine 20mg was decided on in order to reduce dysesthesia but was unable to be continued because it caused drowsiness in the patient. At that time no new analgesic therapy was started due to the improvement of somatosensitive symptoms reported by the patient.

**Figure 2:**
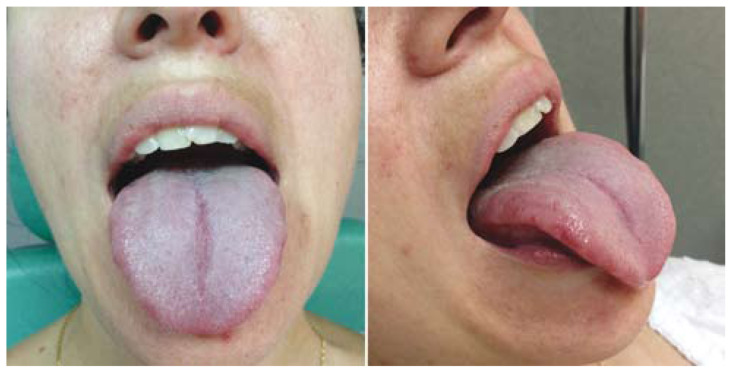
6th month post-removal. A decrease in fungiform atrophied area was observed despite the persistence of bites signs.

After a further follow-up at 6 months the patient reported a great improvement in somatosensory symptoms. Physical examination showed a clear decrease in lingual atrophy of fungiform papillae, although signs of recent bites persisted on the right tongue side (Fig. 2).

## Discussion

The lingual nerve injury, temporary or permanent, as a result of third molar extraction is unpredictable and its cause is controversial (13,20,21,22,23).

There are multiple risk factors that preclude the surgeon from being able to predict or control this complication because of its variable anatomy. Different studies have linked the lesion of the lingual nerve to lingual flap retraction (24), lingual cortical plate trauma during the osteotomy or odontosection, supracrestal incision, duration of the intervention, the depth of the suture, anesthetic block (25) (two times more frequent than the inferior alveolar nerve injury), and even the type of third molar angulation (more frequent in disto-angular and horizontal) (26).

Taking into account the factors involved here, the most likely cause of complication observed in our patient could be related to any of the following facts. One of them could be the individual anatomical variability of the lingual nerve that may result in an injury despite extreme caution during the extraction. Troncular anesthetic block, either by direct nerve injury or anesthetic neurotoxicity, may also be involved. Some authors recommend inserting the needle parallel to the medial mandibular branch to get a lateral relationship with the lingual nerve during the blockade in order to reduce the number of direct nerve injuries and/or anesthetic intrafascicular infiltration (25). Likewise, the depth and/or location of the suture used to close the wound may cause lingual nerve entrapment or direct injury. Finally, the disto-angular position involved the need for a distal osteotomy and lingual flap elevation to perform the extraction properly.

The persistence of fungiform papillae atrophy is an important severity indicator of the nerve injury (28,29). This alteration usually improves slowly during the first 6 months postoperatively. The lack of recovery in this period of time or the duration of symptoms beyond 2 years is a sign of a bad prognosis and it makes unlikely a spontaneous somatosensory recovery (15). In these cases you can opt for clinical observation with drug treatment (30) (tricyclic antidepressants or carbamazepine). Microsurgical reconstruction can be considered in permanent injuries with severe dysesthesia but no significant differences have been demonstrated between conservative vs. surgical treatment as the best therapy option (31).

## Conclusion

The lingual nerve injury after third molar removal is an important complication to consider before subjecting the patient to the intervention due to its remarkable incidence, its unpredictable cause and the significant discomfort it can generate. Depending on the severity or chronicity of the injury, everyday life can be altered considerably. It is therefore vital to know the different risk factors involved in order to minimize the possible damage and report in detail to the patient before surgery to avoid medico-legal issues.
